# Validation and reliability testing of a new, fully integrated gait analysis insole

**DOI:** 10.1186/s13047-015-0111-8

**Published:** 2015-09-22

**Authors:** Benedikt Johannes Braun, Nils Thomas Veith, Rebecca Hell, Stefan Döbele, Michael Roland, Mika Rollmann, Jörg Holstein, Tim Pohlemann

**Affiliations:** Department of Trauma, Hand and Reconstructive Surgery, Saarland University, Building 57, Kirrbergerstr. 1, 66421 Homburg, Germany; BG Trauma Center, Department of Trauma Surgery, Eberhard Karls University Tübingen, Tübingen, Germany; Saarland University, Chair of Applied Mechanics, Saarbruecken, Germany

**Keywords:** Gait analysis, Integrated insole system, Validation, Reliability

## Abstract

**Background:**

A new tool (OpenGo, Moticon GmbH) was introduced to continuously measure kinetic and temporospatial gait parameters independently through an insole over up to 4 weeks. The goal of this study was to investigate the validity and reliability of this new insole system in a group of healthy individuals.

**Methods:**

Gait data were collected from 12 healthy individuals on a treadmill at two different speeds. In total, six trials of three minutes each were performed by every participant. Validation was performed with the FDM-S System (Zebris). Complete sensor data were used for a within test reliability analysis of over 10000 steps. Intraclass correlation was calculated for different gait parameters and analysis of variance performed.

**Results:**

Intraclass correlation for the validation was >0.796 for temporospatial and kinetic gait parameters. No statistical difference was seen between the insole and force plate measurements (difference between means: 36.3 ± 27.19 N; *p* = 0.19 and 0.027 ± 0.028 s; *p* = 0.36). Intraclass correlation for the reliability was >0.994 for all parameters measured.

**Conclusion:**

The system is feasible for clinical trials that require step by step as well as grouped analysis of gait over a long period of time. Comparable validity and reliability to a stationary analysis tool has been shown.

## Background

Gait analysis is a quick and powerful tool with a wide range of clinical applications in various fields [1, 2]. However, due to the expensive and highly specialized equipment required, gait studies are mostly limited to academic research centers and small sample sizes [3] and no large-scale, randomized controlled trials have been performed [4]. Several authors have proposed inexpensive accelerometer-based systems to remedy this situation [5, 6]. Through mathematic transformation they adequately measure step time and length [7]. With these systems however only temporospatial gait parameters can be recorded; kinetic gait parameters, such as ground reaction force, cannot be measured [5]. As these kinetic parameters are important for clinical studies, especially in fracture [8] and rehabilitation research [9] different methods are needed.

Apart from the fact that its availability is mainly limited to research centers, conventional gait analysis is further hindered by its stationarity and that it only allows momentary views of the patient’s gait in a confined research environment. Even smaller, wearable systems have to be attached to an external apparatus, or are limited by their battery capacity, data storage and other device specific factors [3, 10, 11]. Furthermore, the use of these systems is at an early clinical stage and their full potential not yet developed [12]. As most disease processes are continuous, tools with long-term, continuous measuring capabilities are needed. For this reason a new pressure-measuring insole with built in battery and data storage was developed in cooperation with the AO Foundation (AO Foundation, Davos, Switzerland). The system offers complete independence from any external measures for up to 4 weeks and monitors a patients every step during this time. It is currently in preliminary research and clinical use in rehabilitation, neurology and orthopedic trauma.

In order to establish this new continuous gait analysis system as a research tool thorough testing of the new device’s validity and reliability is necessary before clinical trials are feasible. The purpose of the current study is to show the validity and reliability of a new and promising continuous gait analysis tool—the OpenGo Sensor Insole (Moticon GmbH).

## Methods

### Participants

Twelve healthy individuals between the age of 18 and 37 took part in the study. None of the participants had any history of physical or neurological conditions which might interfere with their respective gait. All participants gave written informed consent. Ethical approval was granted by the local ethics committee.

### Insole

**Fig. 1:**
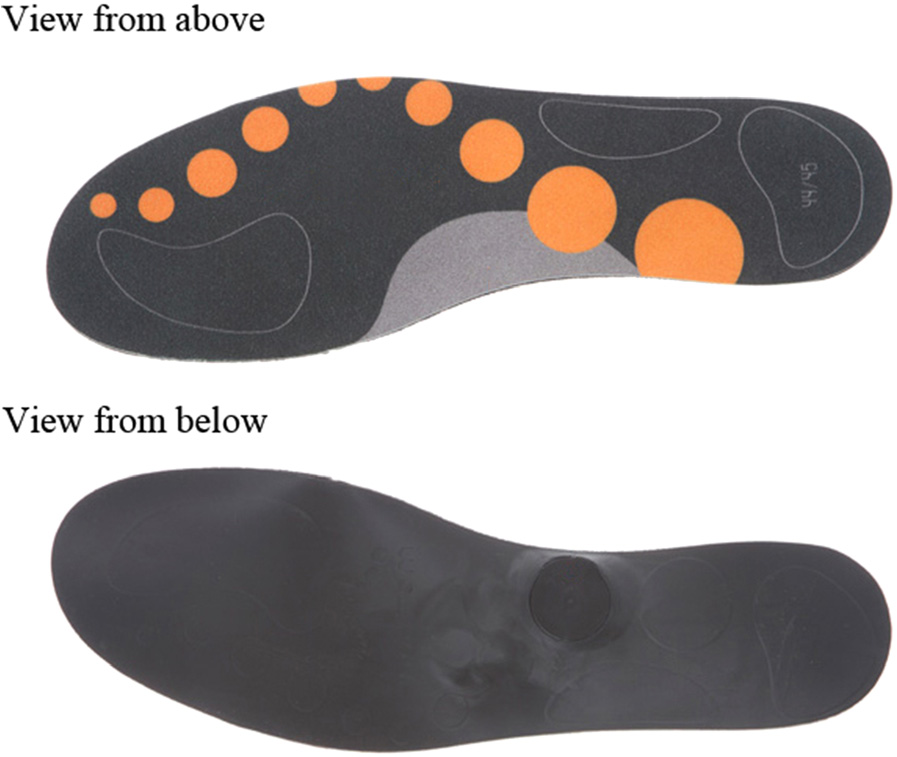
Standard right OpenGo insole. Artificial leather cover. 13 capacitive pressure sensors, accelerometer and thermometer. The round opening for a regular 3,7 V Li-ion battery can be seen from the view from below

The insole weighs no more than 80 grams and looks and feels like a regular insole worn by runners for extra cushioning (Fig. 1). It incorporates 13 capacitive pressure sensors, a 3D accelerometer, as well as a temperature sensor. It measures peak pressures, pressure distribution, acceleration, motion sequences, gait patterns and temperature.

The insole operates completely wireless and only needs to be activated once by a study nurse. It runs for approximately up to 4 weeks on a single battery charge and data are stored on an incorporated flash storage.

The patient is free to remove the sole from his or her shoe and place them in any other shoe at any time. The top layer of the sole is washable and desinfectable.

### Experimental protocol

Every participant performed 3 walks on a standard treadmill at 2 different speeds: 1,0 m/s and 1,7 m/s. Each walk lasted 3 min. Gait was continuously sampled by the Moticon insole at 50 Hz. Gait data were automatically segmented and analyzed by the proprietary Beaker software (version 01.01.14).

Validation of the insole system was performed with the FDM-S pressure plate (Zebris Medical GmbH) [13, 14]. It incorporates 2560 sensors on an area of 54 by 34 cm, giving a resolution of approximately 8.7 sensors per square inch. Reported accuracy is within 5 %.

Each participant performed 30 steps with the dominant leg on the pressure plate at a preferred normal gait speed with 10 m gait in advance. The insole values were measured simultaneously under shod conditions and matched to the corresponding force plate steps. Gait data were sampled at 50 Hz. Segmentation and analysis was performed with the commercially available WinFM software (Zebris Medical GmbH). Resulting forces and contact times between simultaneous steps on the pressure insole and force plate were compared.

### Statistical analysis

Mean and standard deviation were calculated independently for every gait parameter measured at each speed and for every trial. Gait data were screened for normality with the Shapiro-Wilk-Test. ANOVA with a Bonferroni posttest and *t*-Test were performed to compare the gait parameters of the three treadmill trials, as well as left and right foot values. Intraclass correlation coefficients were calculated for the validation and reliability of the trials. *P* < 0.05 was defined as statistically significant.

## Results

**Fig. 2:**
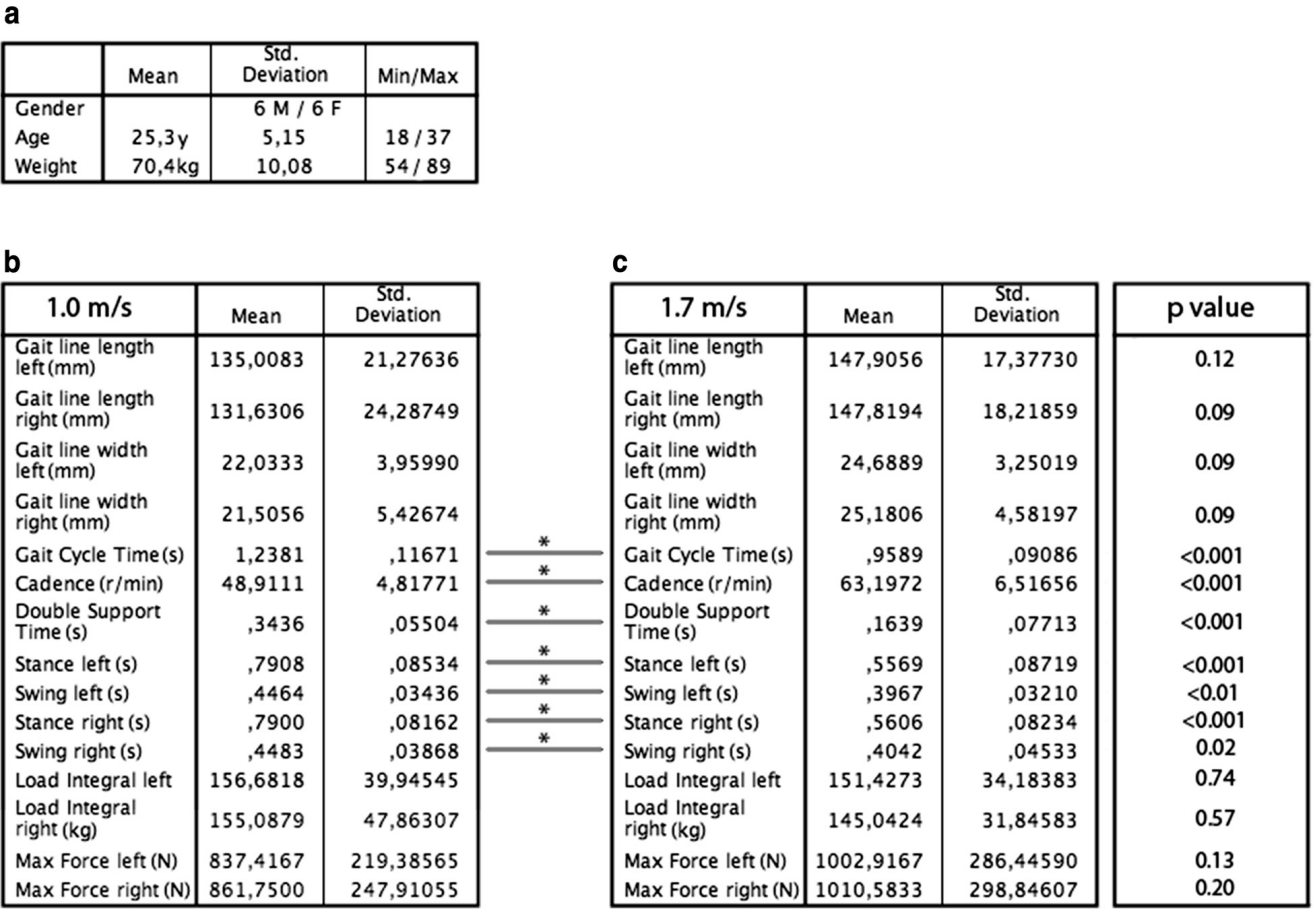
**a** Descriptive statistics of the participants. Average gait parameters as measured by the OpenGO insole at 1.0 m/s (**b**) and 1.7 m/s (**c**) are shown. * < 0.05

Figure 2 shows the participants characteristics and average gait parameters measured via the insole for every trial and gait speed. There were no statistically significant differences between the trials for any gait parameter within each gait speed group (1.0 m/s: *p* = 0.99; 1.7 m/s: p > 0.99). No statistically significant difference between the left and right foot was seen (1.0 m/s: *p* = 0.46; 1.7 m/s: *p* = 0.92). In a comparison of the two speeds there were statistically significant differences in gait cycle time (difference between means: 0.28 ± 0.04 s; *p* < 0.001), cadence (difference between means: 14.29 ± 2.34 r/min; *p* < 0.001), double stance time (difference between means: 0.18 ± 0.03 s; *p* < 0.001), left and right swing (difference between means: left: 0.05 ± 0.01 s; *p* < 0.01; right: 0.05 ± 0.02 s; *p* = 0.02) and stance time (difference between means: left: 0.16 ± 0.31 s; *p* < 0.001; right: 0.16 ± 0.3 s; *p* < 0.001).

### Validity

**Fig. 3:**
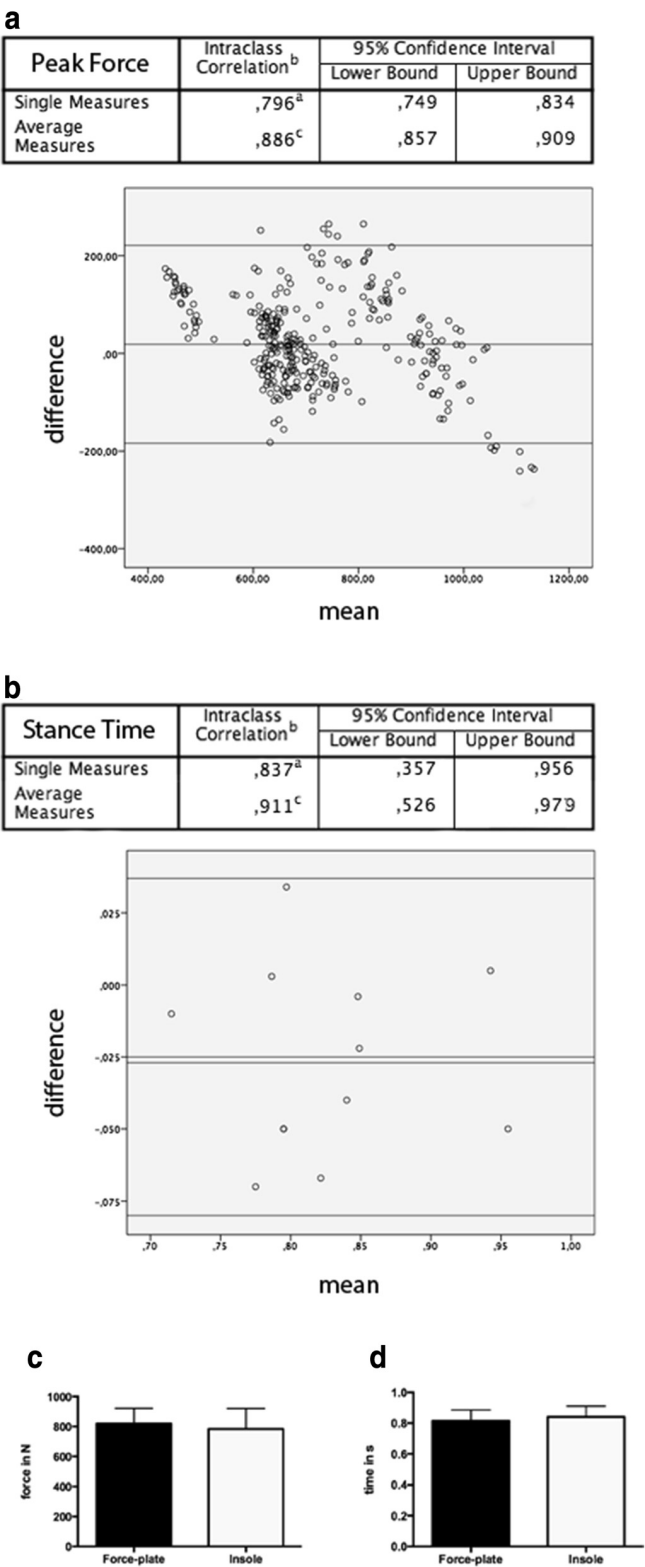
Validation results for peak force (**a**) and stance time (**b**) measurements. The intraclass correlation coefficients for single, as well as average measures are shown. The corresponding Bland-Altman plot is shown beneath each table. Bar graphs with standard deviation for resultant force (**c**) and step time (**d**) are shown. No significant differences between both systems for resultant force (difference between means: 36.3 ± 27.19 N; *p* = 0.19), as well as stance time (difference between means: 0.027 ± 0.028 s; *p* = 0.36) were seen. Data from 360 steps over the force plate are shown

The intraclass correlation was calculated between the average measures of thirty steps on the Zebris pressure plate, as well as thirty corresponding steps with the OpenGo insole worn simultaneously. Intraclass correlation (ICC 3.1/k) for the stance time in seconds was 0.837 and for the resultant force measurements 0.796 for single measures and 0.911/0.886 for average measures (Fig. 3 a, b). The corresponding Bland-Altman plots show over 95 % of the values between the limits of agreement (a = 0.05) and similar error margins for both tests. No statistically significant difference was seen between both systems for resultant force (difference between means: 36.3 ± 27.19 N; *p* = 0.19), or for stance time (difference between means: 0.027 ± 0.028 s; *p* = 0.36) (Fig. 3 c). The data were calculated from 360 steps on the force plate with the insole worn simultaneously under shod conditions.

### Reliability

**Fig. 4:**
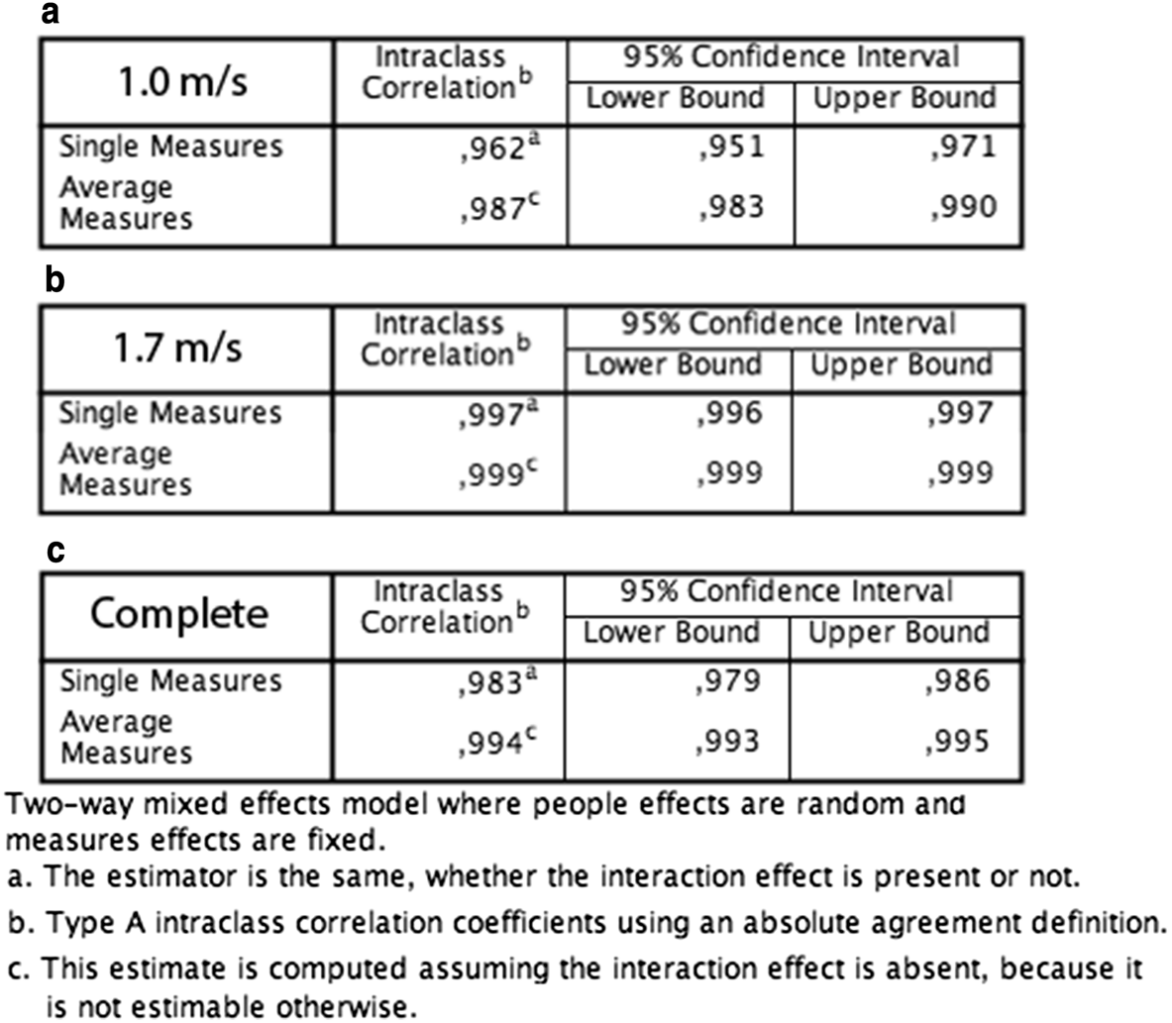
Retest reliability calculations for trials at 1.0 m/s (**a**), 1.7 m/s (**b**) and both combined (**c**). In all 356 Measurements comprising of over 10000 steps were compared

Intraclass correlation (ICC 3,1/k) between each item in every retest was 0.983 for single measures and 0.994 for average measures (Fig. 4 c). Results from the two different gait speeds are shown in Figs. 4a and b, respectively. In all, 356 different measurements comprising over 10000 single steps under shod conditions were used for the calculations.

## Discussion

Modern gait analysis offers excellent opportunities for identifying pathological gait in many clinical fields: the measurement of subtle gait changes in Parkinson patients, the tracking of recovery in knee arthroplasty patients, the monitoring of various lower extremity fractures and many more [1, 2, 8, 12, 15, 16]. These studies highlight the great potential of gait analysis, whilst revealing its momentary weaknesses. Many different gait analysis systems exist, but their use is restricted mainly to academic research centers [5], considerably limiting their potential efficacy [4]. Furthermore, since only momentary measurements are performed, pathologies are mostly detected at a later point in time when preventive measures are limited and clinical interventions often needed [3, 11, 17, 18].

Especially in trauma care, where fracture healing is influenced by the biomechanical environment as soon as treatment is initiated, early, continuous application of these gait analysis tools is the key to influencing the healing process [19–21]. This was one of the underlying reasons for the development of the insole now under examination. It measures and stores every gait event over 4 weeks completely independent of any other external measures. Analysis is fully automatic: either as a step-by-step analysis or as a grouped analysis showing the daily and weakly activity level. The insole also generates raw data that are available for further segmentation and analysis. Unlike other wearable sensor systems, this insole has the advantage of being fully integrated into the sole and does not require any external measurements during the 4 week period [22].

In order to accommodate all the systems in the insole and to enable the independent running time of up to four weeks, fewer sensors are used than in other available in-shoe systems [23–25]. However, despite the reduced sensor capability, the validation values presented in this study show a good correlation between the insole and the force plate system for temporospatial (ICC > 0.837 stance time) and kinetic parameters (ICC >0.796 peak force). Furthermore, there was no statistical difference between the insole and the force plate with regard to the resulting force and stance time. The Bland-Altman plots confirm the validation results, showing equal error margins between the limits of agreement. All of these are well within the level of ICC values for established gait analysis systems [26–28].

The retest reliability values show excellent concordance (ICCs > 0.983) between the three trials, not only confirming the insole’s reliability but also the reliability of the automated analysis process. The automated analysis process is a key factor of the proposed system, since its ease of use aims at increasing applicability, especially in smaller clinics without academic research centers. The reliability values for this system are in accordance with other reported, wearable systems [5]. Furthermore the established gait characteristics and significant differences between the chosen gait speeds are within the known ranges for other validated and established systems [29]. The standard deviations and variances are also within these limits, further confirming the insole’s validity. Step by step reliability analysis and dominant foot validation were performed in this study to account for any potential left to right variability [30].

### Limitations

This study has several limitations, one being the overall low number of participants. This problem was addressed by performing the study as a step-by-step analysis, amounting to over 10000 measured steps. Furthermore, reliability testing was conducted under standardized treadmill conditions, limiting its transferability to overground conditions. Validation measurements were performed simultaneously under shod conditions, creating a foot-sole interface and a shoe-force plate interface. For this reason only resultant forces were compared. No damping effect of the shoe’s insole was observed. Measurements under unshod conditions are not possible with the insole system. Further studies on the influences of different footwear on the insole measurements and long-term reliability testing under overground conditions are necessary.

## Conclusion

This study shows the within test reliability and validity of a new, fully integrated gait analysis tool. The system can be used in broad clinical trials that require step by step as well as grouped analysis of gait over a long period of time with the validity and reliability of a stationary analysis tool. Combined with the reliable automated analysis system, broad applicability outside of academic research centers is feasible.
